# Development and Future Trends of Protective Strategies for Magnesium Alloy Vascular Stents

**DOI:** 10.3390/ma17010068

**Published:** 2023-12-22

**Authors:** Dexiao Liu, Ke Yang, Shanshan Chen

**Affiliations:** 1 Shi-Changxu Innovation Center for Advanced Materials, Institute of Metal Research, Chinese Academy of Sciences, Shenyang 110016, China; 2 School of Materials Science and Engineering, University of Science and Technology of China, Shenyang 110016, China

**Keywords:** magnesium alloy, cardiovascular stent, corrosion resistance, coatings, rapid endothelialization

## Abstract

Magnesium alloy stents have been extensively studied in the field of biodegradable metal stents due to their exceptional biocompatibility, biodegradability and excellent biomechanical properties. Nevertheless, the specific in vivo service environment causes magnesium alloy stents to degrade rapidly and fail to provide sufficient support for a certain time. Compared to previous reviews, this paper focuses on presenting an overview of the development history, the key issues, mechanistic analysis, traditional protection strategies and new directions and protection strategies for magnesium alloy stents. Alloying, optimizing stent design and preparing coatings have improved the corrosion resistance of magnesium alloy stents. Based on the corrosion mechanism of magnesium alloy stents, as well as their deformation during use and environmental characteristics, we present some novel strategies aimed at reducing the degradation rate of magnesium alloys and enhancing the comprehensive performance of magnesium alloy stents. These strategies include adapting coatings for the deformation of the stents, preparing rapid endothelialization coatings to enhance the service environment of the stents, and constructing coatings with self-healing functions. It is hoped that this review can help readers understand the development of magnesium alloy cardiovascular stents and solve the problems related to magnesium alloy stents in clinical applications at the early implantation stage.

## 1. Introduction

**Figure 1:**
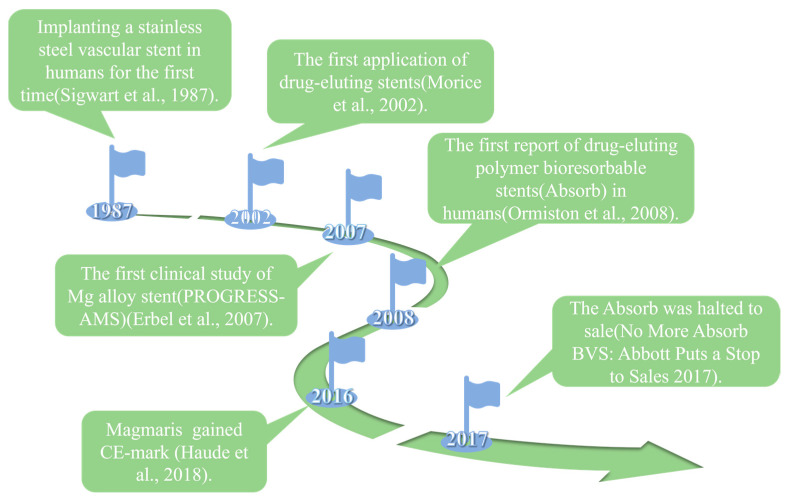
Timeline of progress in the development of stents [3,7,11,12,13,14].

Cardiovascular disease (CVD) takes the lives of around 17.9 million people annually and stands as the primary cause of death globally [1]. One of the rapidest and most effective medical procedures for treating coronary and peripheral artery disease is to use vascular stents [2]. The entire evolution of vascular stents goes through three generations: the first-generation bare-metal stents, the second-generation drug-eluting stents, and the third-generation biodegradable drug-eluting stents (Figure 1).

The bare-metal stents (BMS) were invented and used in clinical settings first. In 1987, the clinical implantation of stainless steel stent into a human coronary artery was first reported [3]. BMS could provide arterial support and significantly improve the clinical and angiographic outcomes in patients [4]. However, implantation of bare-metal stents (BMS) might result in symptoms including in-stent restenosis (ISR) and stent thrombosis (ST) [5,6]. To address the issues of ISR and ST associated with BMS, drug-eluting stents (DES) were created. These stents are coated with polymer coating to load and deliver drugs, such as sirolimus and paclitaxel [7,8]. However, as a permanent implant in the vasculature, DES still brought the potential risks of thrombogenicity, delayed re-endothelialization, mismatches in mechanical behavior, long-term endothelial dysfunction, and chronic local inflammatory reactions [9,10].

To construct innovative stent systems to resolve the above clinical issues, the biodegradability and biocompatibility advantages of bioabsorbable polymer and metal stents have gained attention for application [15]. The most commonly used bioabsorbable polymer material is poly-L-lactic acid (PLLA), and the PLLA-based bioabsorbable polymer stents have achieved promising clinical results. The bioabsorbable everolimus-eluting stent system (Absorb; Abbott Vascular, Santa Clara, CA, USA) is produced using bioabsorbable polylactic acid as a backbone and coated with a more rapidly absorbed polylactic acid layer carrying the antiproliferative drug everolimus (Novartis, Basel, Switzerland) [11]. The bioabsorbable polymer stents were developed to offer similar radial strength and anti-restenosis effectiveness in comparison to the metallic drug-eluting stents [16]. Meanwhile, the long degradation cycle of PLLA stents did not match the healing characteristics of the implant site [2]. The issues of a reduced stent area and stent malapposition are challenges for the polymeric stent, which may initially lack sufficient radial force, resulting in subsequent recoil and malapposition of the stent as bioabsorption is further decreased [17]. Due to the lower radial strength, a thicker strut size is necessary for these polymer stents compared to the majority of metal stents. Clinical trials have demonstrated that the bioabsorbable everolimus-eluting Absorb stents were associated with increased rates of both device-oriented and patient-oriented composite endpoints at 2 years postoperative follow-up [18]. The leading mechanism underlying very late stent thrombosis was the stent discontinuity, which indicated an adverse resorption-related process, followed by malapposition and neoatherosclerosis [19]. Abbott Vascular called a halt to sales of the Absorb bioabsorbable vascular stent on 14 September 2017 [12].

Degradable metal stents possess higher mechanical strength and biocompatibility compared to polymer stents. The bioabsorbable metal stents include magnesium alloy stents, iron alloy stents and zinc alloy stents. The iron alloy stent possesses good mechanical properties, a high elastic modulus and high radial strength, which can well satisfy the mechanical requirements of a stent. Meanwhile, the hemolysis rate of pure iron is less than 5% and there is no sign of thrombosis, indicating that the iron-based stent has good hemocompatibility [20]. However, the complete biodegradation time of the iron alloy stent is still too long as it far exceeds the time required for arterial remodeling and healing (6–12 months) [21]. In general, the alloy corrodes slowly for stenting applications and accumulates a large amount of corrosion product that repels surrounding cells and biological matrix. Moreover, it is neither excreted nor metabolized promptly. Therefore, future research on iron alloy stents should focus on improving the absorption, conversion, metabolism, and elimination of the degradation products [22]. As an essential element in the human body, zinc has a suitable degradation rate, making it one of the choices for biodegradable metal stents [23,24]. The present results revealed that there was no stent thrombosis in a preclinical study. However, the zinc-based stent exposed a significant “strain-softening” effect that may result in limited uniform deformation and possible unexpected failure [22]. Currently, there have been attempts to solve the strain softening problem through some material processing techniques, such as equal-channel angular pressing.

Magnesium alloy stents were the first biodegradable metal stents to have been used in clinical applications due to their outstanding biocompatibility, biodegradability and mechanical properties. Mg alloy stents could be degraded and completely absorbed in vivo, thus avoiding the neointimal hyperplasia and stenosis caused by a long-term foreign body reaction. Mg alloys allow for faster expansion and have more consistent mechanical strength and ductility than bioabsorbable polymer stents. This improves radial support for vessels and makes stent processing and crimp–expansion deformation easier [25]. However, the rapid degradation and localized degradation of Mg alloy stents constrain their clinical application, and rapid degradation may result in restenosis and a loss of mechanical integrity. Based on the computational modelling of the corrosion process, it was found that heterogeneous corrosion leads to a significant reduction in the supporting performance of stent, but a slight reduction in mass loss compared to homogeneous corrosion [26]. In addition, the degradation of magnesium alloys could cause an elevation of the local pH and an accumulation of hydrogen, which may lead to the obstruction of blood circuits and tissue necrosis [27]. Rapid degradation can generate high concentrations of Mg ions in a local environment. These ions have the potential to cause coagulation or inflammation and hinder the process of vascular remodeling [28]. Therefore, it is very important to effectively regulate the corrosion behavior of magnesium alloys to promote the clinical application of magnesium alloy stents. This is also the key research issue for biodegradable magnesium alloy stents at present.

To solve this bottleneck problem of the rapid degradation of magnesium alloy stents, many studies have been conducted on the material composition, design optimization for stent and coatings of magnesium alloys, etc. These studies have promoted the forward the development of magnesium alloy stents to various degrees. The first application of a biodegradable magnesium alloy stent was a milestone in time [29], marking the incorporation of biodegradable vascular stents into the time coordinates of stent development. Late postoperative follow-up showed that the magnesium alloy stent exhibited certain advantages over the bioabsorbable PLA stent. However, a relatively high incidence of the device-oriented composite endpoint (DOCE) was found during follow-up [30].

**Figure 2:**
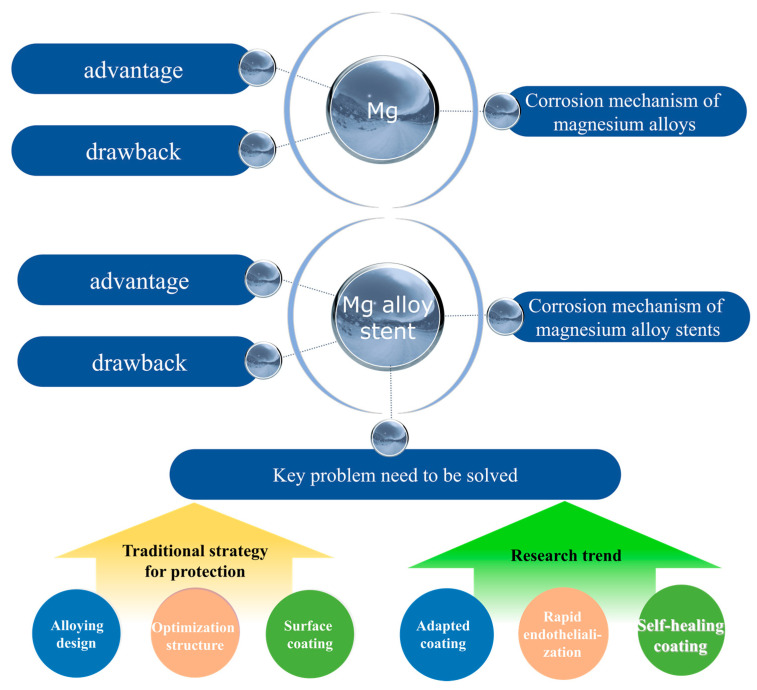
A comprehensive summary of the review.

This article reviews the development history of magnesium alloy stents, summarizes the protection strategies for the stents, and prospects the future development trends of stent protection (Figure 2). Focusing on solving the problems of rapid degradation and short-term strut fracture of magnesium alloy stents, unique strategies are discussed and proposed, which are expected to create new ways to reduce the degradation rate of magnesium alloys and then improve the comprehensive performance of magnesium alloy stents.

## 2. Development History of Magnesium Alloy Stents (BIOTRONIK)

**Figure 3:**
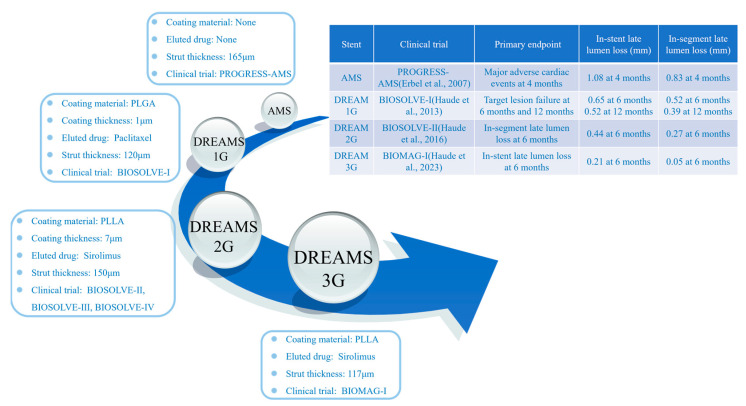
The characteristics of biodegradable magnesium alloy stents made by Biotronik [13,32,33,34].

BIOTRONIK in Germany is dedicated to the research and development of biodegradable magnesium alloy stents and has developed several generations of products, including Lekton Magic (AMS), DREAMS 1G, Magmaris (DREAMS 2G), and DREAMS 3G, achieving good results in research [31,32], as shown in Figure 3. 

The material of the first reported biodegradable magnesium stent was AE21 (containing 2% aluminum and 1% rare earths), which was implanted into the coronary arteries of domestic pigs in 2003. There was no stent that experienced major problems or initial signs of fracture during implantation, and no significant thrombosis occurred [29]. Waksman et al. [35] compared the safety and efficacy of biodegradable magnesium alloy stents with stainless steel stents in porcine coronary arteries. At 28 days, the magnesium alloy stent began to show signs of degradation. At 28 days and 3 months, the magnesium alloy stent had a significantly smaller area of neointima than the stainless steel stent. However, the reduction in neointimal formation did not translate into a larger vessel lumen. This could be caused by under-expansion of the stent during deployment, early or late recoil, or potentially a combination of both. Within 3 months, the magnesium alloy stents had completely degraded or were in an advanced stage of degradation in porcine vessels. There was a modest degree of neointimal formation, which remained steady between 30 and 90 days. This indicated that the primary reason for restenosis after the implantation of biodegradable magnesium alloy stents was an early recoil. This issue could be solved by slowing down the pace of stent degradation [36].

As the first biodegradable magnesium alloy stent, a Lekton Magic (Biotronik, Bulach, Switzerland) stent was implanted in 20 patients for the treatment of critical lower limb ischemia [37]. No adverse events were reported during the procedures. Postprocedural color Doppler flow and magnetic resonance demonstrated accurate positioning and expansion of the stents and no early recoil. At 1 month postoperation, no patient showed any symptom of allergic or toxic reactions to the stent material. The primary clinical patency rate at 3 months postoperatively was 89.5%. All patients had no amputation and the limb retention rate was 100% [38]. Afterwards, the stent was evaluated in human coronary arteries by the PROGRESS-AMS study (Clinical Performance and Angiographic Results of Coronary Stenting with Absorbable Metal Stents) [13]. It was demonstrated that in atherosclerotic coronary arteries, biodegradable magnesium alloy stents could be delivered and expanded at high pressure, furnishing reliable mechanical support and accomplishing a lumen enlargement comparable to the immediate lumen gain attained with customary metal stents. The absence of raised creatine kinase (CK) levels and ischemic episodes suggested that stent material embolization is absent in the early phases of stent degradation, indirectly confirming that the degradation of the alloy occurred within the vessel wall, with rapid re-endothelialization and strut coverage. Angiography at 4 months post-operation indicated an augmentation of the diameter of stenosis of 48.4%. An intravascular ultrasound (IVUS) examination of biodegradable metal stents in human coronary arteries revealed that the early recoil significantly contributed to restenosis within 4 months [39]. 

To extend vessel support and minimize neointimal growth, a biodegradable metal stent has been redesigned and layered with a biodegradable poly(lactide-*co*-glycolide) polymer matrix (PLGA) that contains the anti-proliferative drug paclitaxel [40]. It has comparable efficacy and healing properties to paclitaxel-eluting permanent metal stents in a porcine coronary artery. A multicenter, first-in-human trial (BIOSOLVE-I) of drug-eluting absorbable metallic stents (DREAMS) was undertaken [33]. At 12 months of implantation, DREAMS had a better result of clinical driven target lesion revascularization compared to bare absorbable metal stents (4.7% vs. 26.7%) [13] while maintaining excellent biosafety. The incidence of target lesion failure for DREAMS at the 6- and 12-month periods was comparable to the contemporary drug-eluting stents and the Absorb bioresorbable everolimus-eluting coronary stent systems. Nevertheless, the late lumen loss of DREAMS was not as impressive as those of the other stents.

DREAMS 2G, also known as Magmaris, was the second generation of drug-eluting absorbable metallic stents. It was coated with bioabsorbable poly-L-lactic acid (PLLA) carrying sirolimus. Animal tests indicated that the Magmaris had a favorable safety profile [41]. After 12 months postoperation, DREAMS 2G had almost completely degraded in porcine coronary arteries. Strut discontinuities were uncommon at 28 days and increased rapidly up to 90 days, with almost complete degradation of the stent at 1 year [42].

In order to evaluate the safety and performance of Magmaris in patients with de novo coronary artery lesions, the BIOSOLVE-II and BIOSOLVE-III studies were performed [14,34]. Compared to DREAMS 1G, Magmaris had a more flexible and robust stent design, higher bending flexibility, and higher radial force. Sirolimus-loaded PLLA as a drug-eluting coating was more effective at inhibiting neointima formation. The angiographic performance of Magmaris stents showed that no definite or probable stent thrombosis occurred after 12 months of implantation. The late lumen loss of Magmaris has been improved, maintaining favorable clinical and safety profiles. The 30 days and 1 year follow-up data showed that the primary endpoints (death from cardiac causes, myocardial infarction, and stent thrombosis) as well as the incidence of target lesions were significantly lower for Magmaris than Absorb. Biodegradable magnesium alloy stents have presented a more satisfactory safety profile and more favorable clinical outcomes [43]. Magmaris has gained CE-mark in June 2016 [14]. BIOSOLVE-IV verified the safety and efficacy of Magmaris in a significant sample size of individuals, combined with a highly satisfactory safety profile for up to 12 months in a low-risk cohort [44].

**Figure 4:**
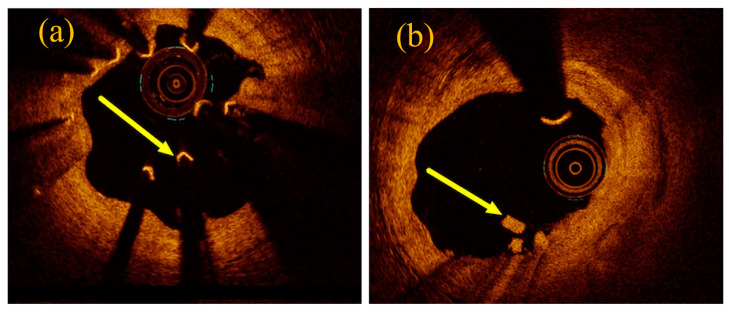
OCT results of Magmaris observed in patients with target lesion failures: (**a**) collapse of the stent and (**b**) uncontrolled dismantling of the device [30].

However, Bossard et al. [30] found a relatively high incidence of device-oriented composite endpoints (DOCE) and even observed stent collapse and uncontrolled dismantling in the follow-up of patients treated with Magmaris. Most adverse events occurred within 24 months of implantation, with target lesion failure (TLF) occurring rarely thereafter. Signs of stent collapse and stent dismantling were found in the long-term follow-up of patients implanted with Magmaris. As shown in Figure 4a, the optical coherence tomography (OCT) image shows a collapsed stent, which appears to be a “free-floating” strut (arrow) that is no longer embedded in the coronary artery wall. In addition, there appears to be restenosis of the inner lumen with significant luminal irregularities and tissue protrusion. Figure 4b demonstrates uncontrolled dismantling of the stent with strut residuals protruding into the vessel lumen (arrows). In addition, the intima appears partially irregular (1–5 o’clock).

Corrosion of the strut is a unique phenomenon of magnesium alloy stents. The corrosion rate of strut may differ among various individuals and may be accelerated by stent malapposition, focal low pH between the vessel wall and the magnesium alloy stent struts and by substantial hinging forces [45]. The rapid degradation rate with a loss of radial strength, combined with a potential accelerated healing response at the dilated stenosis, might contribute to the occurrence of restenosis [46]. A certain degree of late stent disruption might also lead to the development of restenosis [47].

The development of the third generation of drug-eluting magnesium alloy stents (DREAMS 3G) has used a new magnesium alloy, which endows the stent with higher mechanical properties and thinner struts while maintaining the same resorption time. A new marker concept and an increased size portfolio were adopted by DREAMS 3G, which showed favorable safety and performance outcomes comparable to the contemporary drug-eluting stents [32].

## 3. Corrosion Mechanism of Magnesium Alloys and Stents

As a bioabsorbable material device, the degradation of magnesium alloy stents is caused by constant corrosion in the in vivo environment. In order to solve the problems of short-term collapse and rapid attenuation of the support force of magnesium alloy stents in vivo, it is necessary to investigate the most direct reasons leading to the corrosion and fracture of magnesium alloy stents from the corrosion mechanisms of magnesium alloys and stents in order to find effective ways to solve the problems fundamentally.

### 3.1. Corrosion Mechanism of Magnesium Alloys

Magnesium (Mg) has advantages such as a low density and high specific strength, but its chemical activity makes it highly susceptible to corrosion, thus limiting its applications in industry, medical devices and other fields. The corrosion of Mg is essentially an electrochemical process in which Mg is oxidized to MgO and Mg(OH)_2_, which is a highly susceptible spontaneous reaction. The reaction is as described in Equation (1):

(1) Mg (s)+12O2(g)→MgO

The generated MgO layer improves the corrosion resistance of Mg in a dry environment. In a moist environment, the layer formed on the Mg surface contains Mg(OH)_2_ and MgO. Due to the decomposition of water molecules to form H^+^ and OH^−^, leading to the surface hydroxylation of the MgO. At lower water vapor concentrations (greater than 1 ppm H_2_O), the reaction of magnesium oxide in the solid state with water may result in the formation of solid magnesium hydroxide [48].

(2) $$O^{2-}\left(\mathrm{surface}\right)+H_{2}O\;\left(g\right)\to 2{\mathrm{OH}}^{-}$$

(3) $$\mathrm{MgO}\left(s\right)+2{\mathrm{OH}}^{-}\to \mathrm{Mg}{\left(\mathrm{OH}\right)}_{2}$$

Liu et al. [49] found that a two-layer film existed on the surface of Mg exposed to water, consisting of an internal MgO layer near the Mg metal and an external porous Mg(OH)_2_ layer. Mg undergoes electrochemical corrosion in an aqueous environment and the overall reaction is as follows:

(4) $$\mathrm{Mg}+2H_{2}O\to \mathrm{Mg}{\left(\mathrm{OH}\right)}_{2}+H_{2}↑$$

The main corrosion products are Mg^2+^, OH^−^, and H_2_. Electrochemical theory shows that the corrosion rate of Mg is determined by anodic and cathodic reactions. The anodic reaction is:

(5) $$\mathrm{Mg}\to {\mathrm{Mg}}^{2+}+2e^{-}$$

Additionally, the cathodic reaction is:

H^+^ + e^−^→12H_2_ (6)

In acidic and neutral aqueous solutions, the Mg surface layer proves inadequate in shielding the metal substrate. When the hydroxide layer dissolves in aqueous solution, the pH of the solution increases, which leads to the precipitation of Mg(OH)_2_. It has been shown that the corrosion mechanism of Mg involves the conversion of the MgO layer’s dissolution into Mg(OH)_2_ precipitation. This results in the reduction of the surface MgO layer thickness, which exacerbates corrosion [48].

**Figure 5:**
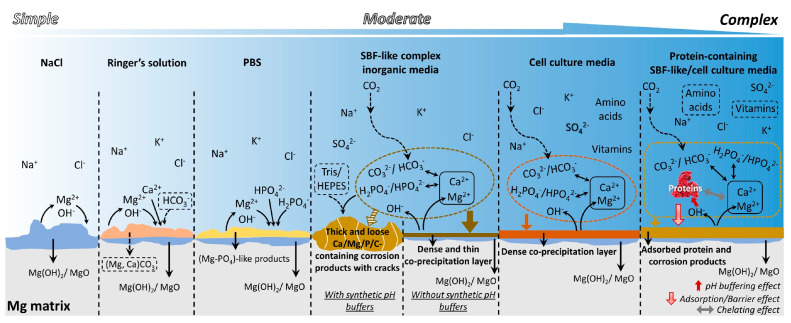
The corrosion behaviors of magnesium in commonly used media [50].

Due to the special application environment of bioabsorbable magnesium alloys, the study of corrosion in vitro is usually carried out in a simulated body fluid environment. Mei et al. [50] discussed the advantages and shortcomings of several commonly used corrosive media for magnesium and their applicability. The corrosion behaviors of magnesium in commonly used media are shown in Figure 5.

Physicochemical characters, such as the impurity content and alloying elements, microstructures (e.g., grain size and second phases, segregation and intracrystalline orientation errors), plastic deformation and internal stress determine the electrode potentials, oxide features and galvanic corrosion tendency in magnesium alloys [51]. Therefore, the corrosion resistance of magnesium alloys can be improved by changing the alloy composition and constituent phases in magnesium alloys, surface coating protection, and adding a corrosion inhibitor.

### 3.2. Corrosion Mechanism of Magnesium Alloy Stents

Compared with the magnesium alloy substrate, magnesium alloy stents not only need to withstand media corrosion but also need to undergo complex deformation during the compression grip–expansion–pulsation process after implantation, and the mechanism of corrosion is more complex. The study found that about 95% of the stent was degraded within 12 months after the implantation of a magnesium alloy stent [52]. The degradation and absorption of the stent occur in two stages. Initially, the magnesium alloy is converted into hydrated magnesium oxide. Subsequently, the magnesium oxide is converted into magnesium phosphate, which is further replaced by amorphous calcium phosphate. Throughout this process, Mg diffuses out of the amorphous matrix and is absorbed by the body, while amorphous calcium phosphate, elements in the alloy, and markers remain in the tissue [34].

#### 3.2.1. Stress Corrosion

The stent should undergo both uniform corrosion and stress corrosion. A corrosion product will form and cover the entire exposed surface of the stent in a “layer-by-layer” pattern during uniform corrosion. However, stress corrosion is prone to occur at places with high residual stress, which would cause the stent ring to shatter quicker than the other locations [53]. Residual stress is generated during the grip and expansion of the stent for implantation, and the presence of stress would accelerate the rate of degradation and the attenuation of the supporting properties [54]. Since the integrity of the stent is usually destroyed first at the location of stress concentration, it can be estimated that stress corrosion should play a major role in stent failure [55].

Aside from the residual stress generated, the coating on the surface of the stent may crack as the stent deforms. Compared to the bare magnesium alloy stents, the HF-treated stents demonstrated exceptional corrosion resistance without deformation. Nevertheless, the surface of the HF-treated magnesium alloy stent showed small fragments and cracks after deformation, which intensified the stent’s corrosion [56]. During the corrosion process, hydrogen may escape locally, pushing away the polymer coating and weakening the adhesion of the outer polymer coating to the stent, which would subsequently accelerate the degradation of magnesium alloy stent [57].

#### 3.2.2. Fatigue

The other reason leading to the premature failure of magnesium alloy stent is the cyclic loading applied by arterial pulses. Magnesium alloys were found to have greater corrosion rates under cyclic loading, and the corrosion rate increases with increasing cyclic loading. In a corrosive environment with cyclic loading, corrosion pits could cause crack initiation with release of the stress concentration [58]. The mechanism of fracture failure was explored by Chen et al. [59]. This study implanted 18 drug-eluting biodegradable AZ31B stents into porcine coronary arteries, showing a large number of fractures leading to the loss of structural integrity. During crack formation, there was no degradation product on the cracks. However, the degradation products on the cracks increased as the implantation time increased. It was inferred that the loss of structural integrity of the AZ31B stent might be the result of stress concentration, degradation and microstructural changes. Shen et al. [60] published the first paper on a numerical simulation of the corrosion fatigue behavior of magnesium alloy stents subjected to cyclic loading in a physiological environment. A numerical continuum damage mechanics model was developed using the finite element method to explain the corrosion fatigue of magnesium alloys and their application in coronary stents. Both corrosion damage and fatigue damage were found to cause the total mass loss of the stent. Fatigue loading or cyclic stress accelerated the degradation of the stent, potentially leading to premature stent failure.

## 4. Traditional Strategy for the Protection of Magnesium Alloy Stents

According to the corrosion mechanisms of both magnesium alloys and stents, different strategies for the protection of magnesium alloy stents were attempted. Alloying is an effective way to improve the mechanical properties of metallic materials, and the addition of alloying elements can affect the corrosion behaviors of materials. Thus, how to balance the mechanical property and corrosion resistance are very important. In addition, design optimization for a magnesium alloy stent could reduce the maximum residual stress by lowering the concentration of residual stress, thereby reducing the degree of stress corrosion. The last and most important strategy is the preparation of a coating on magnesium alloy stents. The coating is a physical barrier against the penetration of corrosive media, and its effectiveness is particularly important for magnesium alloy stents that need to undergo complex deformation.

The three aspects above have improved the corrosion resistance of magnesium alloy stents to a certain extent. Next, the related research progress in these three aspects are introduced one by one.

### 4.1. Alloying Design for Magnesium Alloy Stents

**Figure 6:**
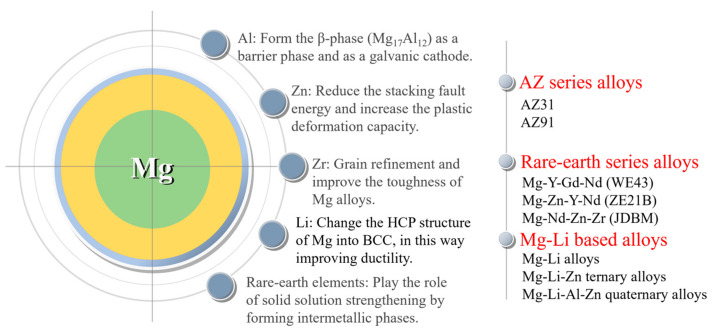
Magnesium alloys for stents and functions of alloying elements.

Commonly employed alloying elements in magnesium alloys consist of basic metal elements such as Al, Zn, Mn, Zr, and Li, as well as rare earth elements; each alloying element has its own effect. Aluminum (Al) is a crucial element in magnesium alloys as it enhances their corrosion resistance and strength. Aluminum can form the β-phase (Mg_17_Al_12_) in magnesium alloys. Increasing its content in the basal phase facilitates the creation of a highly corrosion-resistant surface layer with a substantial aluminum content [61]. As the aluminum content increases, the β-phase increases and more β-phase precipitates along the grain boundaries, protecting the magnesium alloy matrix. By micro-alloying zinc (Zn) in the magnesium matrix, there is a decrease in the stacking fault energy and an increase in the plastic deformation capacity by effectively activating the non-basal slip. Zirconium (Zr) acts as a significant refining agent for the grains and elevates the toughness of magnesium alloys [28]. The Mg-Li (lithium) alloy is a newly developed system with ultra-high ductility and potential applications in vascular stents. However, the presence of Li causes a reduction in the mechanical strength of the alloy. Rare earth elements are added into magnesium alloys and can play the roles of solid solution strengthening by forming intermetallic phases and improving the corrosion resistance of magnesium alloys. Based on the specific alloying effects of each alloying element, three series of magnesium alloys have been attempted to be applied to the vascular stent at present. They are AZ series alloys (AZ31 and AZ91), rare earth series alloys (WE43 [37], Mg-Zn-Y-Nd (ZE21B) [62], and Mg-Nd-Zn-Zr (JDBM) [63]), and Mg-Li series alloys [64] (Figure 6).

The effects and existing problems of different series of magnesium alloys used for vascular stents are reviewed below. AZ series magnesium alloys are a traditional commercially used material that possess excellent and stable comprehensive properties. In a previous study [59], 18 drug-eluting biodegradable AZ31B alloy stents were implanted into porcine coronary arteries, and there were a large number of fractures formed on the stent strut, leading to a loss of structural integrity. Despite some recoil and lumen loss, the lumen remained unobstructed after 3 months of implantation. The occurrence of fracture was related to the strong stress corrosion of the alloy. Yuen et al. [65] investigated the acceptable masses of ten magnesium alloys as degradable biomedical implant materials in their study. The results indicate that aluminum components are usually the least tolerated, and the acceptable mass for Al-containing magnesium alloys is around or below 1 g per person per year, while the limit for other magnesium alloys can exceed 10 g. It means that the element Al has a certain degree of toxicity and needs to be avoided or used with caution.

Hence, rare earth series magnesium alloys were designed and prepared, including WE43 (used as the first-in-men magnesium alloy stent by Biotronik), the Mg-Zn-Y-Nd (ZE21B) alloy developed by Zhengzhou University, China, and the Mg-Nd-Zn-Zr (JDBM) alloy developed by Shanghai Jiao Tong University, China.

The Mg-Y-Gd-Nd alloy (WE43) is a commonly used material for manufacturing biodegradable magnesium alloy stents, and Y (yttrium) and Nd (neodymium) as alloying elements can improve the corrosion resistance of the alloy. Meanwhile, studies have shown that Y, Nd, and Gd (gadolinium) are not cytotoxic [66]. The WE43 alloy stent was implanted in patients for the treatment of severe lower limb ischemia [37]. No adverse events were reported during the procedure, and no patients showed any symptoms of allergic or toxic reactions to the stent material. Magmaris (formerly known as DREAMS 2G), based on the WE43 alloy, is a balloon-expandable, sirolimus-eluting, biodegradable metal stent mounted on a rapid exchange delivery system [52]. This was the pioneer product for biodegradable metal stents, which received CE approval in June 2016. It has exhibited promising clinical outcomes so far.

The Mg-Zn-Y-Nd alloy (ZE21B) was developed with low levels of zinc and yttrium by Zhengzhou University, China, in 2010 [62]. Zinc is known to improve the corrosion resistance of magnesium alloys, leading to the cast Mg-Zn-Y-Nd alloy exhibiting a higher corrosion potential (−1.76 V) compared to the cast Mg-Y-Gd-Nd alloy (−1.95 V). The Mg-Zn-Y-Nd alloy was prepared through a sub-rapid solidification process, exhibiting superior corrosion resistance in a dynamic SBF. The mechanical properties and corrosion resistance of the Mg-Zn-Y-Nd alloy can be further improved using different processing techniques and heat treatments, for example, cyclic extrusion compression (CEC) [67]. The findings demonstrated that the grain size in the alloy was refined to 1 µm with CEC treatment. Furthermore, the second phase distributed in a grid shape along the grain boundaries, and the nanoparticles were uniformly distributed within the grain, leading to a significant improvement in mechanical properties of the alloy. The alloy also displayed uniform corrosion. The corrosion current density of the alloy was observed to decrease from 2.8 × 10^−4^ A/cm^2^ to 6.6 × 10^−5^ A/cm^2^. In 2018, Wang et al. [68] processed tube blanks of the Mg-Zn-Y-Nd alloy using hot extrusion. The annealed and drawn fine tubes exhibited superior mechanical properties. Moreover, the fine tubes demonstrated uniform corrosion in the simulated body fluid and exhibited excellent corrosion resistance. In 2021, Du et al. [69] processed fine tubes of Mg-Zn-Y-Nd alloy using a HTHE (long-time and high-temperature heat treatment, large-reduction-ratio hot extrusion) process, obtaining a refinement of the coarse secondary phase uniformly distributed in the matrix of fine tubes.

The Mg-Nd-Zn-Zr (JDBM) alloy was designed by Shanghai Jiao Tong University (SJTU) for biomedical applications [63]. Nd was used as the main alloying element for strengthening, which was revealed by diminishing the stacking fault energy of the basal plane and producing a pinning effect on the base slip [28]. Additionally, Zr serves as a potent grain refiner in magnesium alloys with better biocompatibility [70]. The extruded JDBM alloy exhibited superior corrosion resistance with slight uniform corrosion. It was found that the corrosion rate of JDBM alloy in Hank’s solution was significantly slow at 0.28 mm/year compared to the AZ31 alloy at 1.02 mm/year [71], which was covered by a more compact and protective layer that prevented the pitting corrosion at the early stage of immersion. Furthermore, bare JDBM stents were implanted into the common carotid artery of New Zealand white rabbits to evaluate the safety, efficacy and degradation behavior of the stent [72]. The results indicated that the bare JDBM stent was effective and safe, and complete re-endothelialization occurred within 28 days. The majority of the JDBM stent struts underwent in situ replacement by degradation products in 4 months.

In addition to these, the Mg-Li alloy was developed through mechanisms such as solid solution strengthening, grain refinement and dispersion strengthening, showing superior corrosion resistance in the meantime. Bian et al. [73] used a high ductility (>40%) Mg-8.5 Li (wt.%) alloy (without rare earth metals and aluminum) to fabricate the stent. A finite element analysis verified the impacts of plastic deformation and residual stress arising from the expansion process on the degradation of this stent. While the stent showed a favorable degradation rate in vitro (0.15 mm/year), different results were found in vivo (>0.6 mm/year). Further, the stents were well-tolerated by the adjacent tissues in pigs, and no thrombosis was reported. Furthermore, a range of Mg-Li based alloys have been developed, for example, Mg-Li-Zn ternary alloy [74] and Mg-Li-Al-Zn quaternary alloy [75]. The current research on Mg-Li alloys in cardiovascular stents mainly focuses on the mechanical properties and corrosion resistance, and the toxicity and biocompatibility still need to be further verified.

Above all, the addition of alloying elements has improved the mechanical properties and corrosion resistance to a certain extent, but it has not well met the clinical demand for magnesium alloy stents. Therefore, other methods should be adopted.

### 4.2. Optimization of Magnesium Alloy Stent Designs

Generally, the radial strength, recoil and non-uniform expansion (a reduced dog-boning effect) during expansion as well as the in-stent restenosis of the stents are influenced by the design of the stents [76]. For magnesium alloy stents, design optimization is another way to improve its corrosion resistance, due to the decrease in the stress concentration and stress corrosion. Finite element analysis (FEA), a simple and efficient method, has been widely used in recent years for the structural design and optimization of magnesium alloy stents. Wu et al. [55] found that the width of the strut was increased by roughly 48%, resulting in improved safety performance (specifically a 29% decrease in maximum principal stress after tissue recoil and a 14% decrease in maximum principal strain during expansion) and a 24% increase in scaffolding ability. The strut width in the final optimized design was not uniform: the mass of the inner straight sections was reduced to a greater extent, while the mass of the outer curved sections was increased. This resulted in reduced maximum strain and a more uniform distribution of strain. Thereafter, a three-dimensional (3D) FEA model combined with a degradable material model was proposed [77], which was used to analyze three different stent designs made from the AZ31 alloy, crimped and expanded in arterial vessels, through the ABAQUS explicit solver. It was verified that the expectation that the design for magnesium alloy stent with more mass and optimized mechanical properties could increase the stenting time. In addition, Wu et al. [53] compared the corrosion resistance of AZ31B alloy stent samples with two designs (an optimized one [55] and a conventional one), which underwent balloon expansion and subsequently immersed in D-Hanks’ solution for a 14-day degradation test. It indicated that the optimized designed stents exhibited superior corrosion resistance to the stents with the conventional design due to less stress distribution in the former ones. The congruity between the numerical simulation and experimental data demonstrated the efficiency of the FEA numerical modelling tool in the design optimization of novel biodegradable magnesium alloy stents.

Increasing the strut size (e.g., a larger strut width) and decreasing the uniformly distributed principal stress are two effective ways of improving the mechanical properties of magnesium alloy stents. Although an augmented strut width could offer adequate radial support to prolong the uniform corrosion time, it might also escalate maximum principal stress during stent expansion and recoil, which could lead to stress corrosion. Degradation failure of magnesium alloy stents typically occurs first at the location of stress concentration. Therefore, the initial phase in designing an optimized stent design is to decrease the maximum principal stress or strain. Conventional stent design usually maintains a constant stent width. For stents with low ductility materials, changing the mass distribution of the stent is also an effective way to optimize the performance of the stent.

### 4.3. Coatings on Magnesium Alloy Stents

Making a coating is an important strategy to improve the corrosion resistance and biocompatibility of magnesium alloy stents. The application of the coating would not bring about any alterations to the microstructure of the magnesium alloy matrix or the stent structure. However, it alters the surface properties, resulting in outstanding corrosion resistance and biocompatibility for the magnesium alloy stent. There have been many kinds of coatings on magnesium alloys. Although a single coating typically does not attain these objectives simultaneously, composite coatings comprising various single-layer coatings can present fresh perspectives for investigations of magnesium alloy stents. When preparing a coating on magnesium alloy stent for surface modification, a chemical conversion film is commonly used to enhance the corrosion resistance of the substrate and improve the adhesion between the substrate and the coating. An outer polymer coating is then prepared to covered the chemical conversion film to further enhance the corrosion resistance and biocompatibility.

#### 4.3.1. Inner Chemical Conversion Coating

Chemical conversion treatment can create a protective layer of metal oxides or other compounds on the surface of magnesium alloys, which acts as a physical barrier to effectively separate the magnesium substrate from the corrosive medium. This treatment improves the adhesion of the final deposited coating to the substrate and then enhances the corrosion resistance and biocompatibility. The commonly used chemical conversion coatings to protect magnesium alloy stents include a micro-arc oxidation coating, phosphate conversion coating, magnesium hydroxide coating and magnesium fluoride coating.

Micro-arc oxidation (MAO), also referred to as plasma electrolytic oxidation (PEO), is an electrochemical process for the controlled oxidation of metal materials to attain surfaces with a particular morphology, thickness, and composition, improving the corrosion resistance and biological properties of the metal materials [78]. The typical MAO coating consists of an inner and outer layer. The compact and uniform inner layer functions as a barrier, impeding the penetration of the solution into the substrate. The presence of oxygen bubbles during the growth of the coating and the thermal stress caused by the quick solidification of the molten oxide in a relatively cold electrolyte could result in a rough outer layer with micropores and microcracks [79]. Therefore, although the inner dense layer of the MAO coating enhances the corrosion resistance, the outer layer permits increased absorption of the corrosive electrolyte into the MAO coating, thus decreasing the coating’s resistance to corrosion [80].

The phosphate conversion coating has shown high biocompatibility, excellent and robust adhesion, a reduced degradation rate and inhibited the negative side effects of magnesium alloy implants in animal models [81]. Phosphate conversion coatings such as magnesium phosphate [82], zinc phosphate [83,84], and calcium phosphate [85,86] have been reported in many studies on the corrosion resistance of magnesium alloys as an environmentally friendly surface modification technique. Zai et al. [87] compared the corrosion resistance and biocompatibility of various phosphate conversion coatings, including magnesium phosphate (Mg-P), calcium phosphate (comprising Ca-P and CaMg-P) and zinc phosphate (comprising Zn-P, ZnMg-P, ZnCa-P, and ZnCaMg-P). Magnesium alloy substrates, as well as magnesium phosphate and calcium phosphate conversion coatings, showed a mixed form of corrosion involving filiform and pitting during extended immersion in Hanks’ solution. Conversely, the primary form of corrosion in the zinc phosphate conversion coating was pitting. ZnMg-P provided superior anti-corrosion performance to the other coatings due to its highly stable structure that effectively inhibited the propagation of filiform corrosion. Based on the results of the cell viability test, the calcium phosphate conversion coating displayed superior biocompatibility compared to zinc phosphate and magnesium phosphate conversion coatings, as well as the bare magnesium alloy substrate. Mao et al. [88] prepared a uniform Mg_3_(PO_4_)_2_ coating on the surface of the JDBM alloy using a chemical transformation method in a mixed phosphate solution of 5% NaH_2_PO_4_ and 3% Na_3_PO_4_ at a ratio of 1:1, which improved both the corrosion resistance and biocompatibility of the alloy. The lamellar-structured phosphate coating exhibited an exceptional affinity for cells by facilitating cell adhesion and spreading. The magnesium phosphate conversion coating was observed to comprise an outer layer generated through precipitation and an inner layer grown in situ [89].

The degradation product of magnesium in the human body, Mg(OH)_2_, exhibits superb biocompatibility without toxicity. The hydrothermal method [90,91] can be used to prepare a uniform and compact hydroxide layer on the surface of magnesium alloys, which has strong adhesion with the matrix and greatly reduces the degradation rate of magnesium alloys. However, as the degradation process of magnesium alloy advances, it leads to the formation of porous Mg(OH)_2_ on the surface. The Mg(OH)_2_ coating contains micro-pores and microcracks that function as transport channels for corrosive media. Therefore, these coatings do not provide long-term and effective protection for magnesium alloys. In order to improve the corrosion resistance of the hydroxide coating, the layered double hydroxide (LDH) coating was further developed. The Mg-Al LDH has been considered an effective agent to retard the corrosion reaction. The atomic structure comprises brucite-like octahedral layers that are positively charged by the substitution of a few Mg^2+^ with Al^3+^ ions [92]. The Mg-Al LDH incorporating carbonate was effective at capturing Cl^−^ anions in a corrosive environment within the LDH interlayer, thus enabling the layer to shield the magnesium alloy from corrosion [93]. The Mg-Al LDH coating on magnesium alloys showed favorable corrosion resistance both in vitro and in vivo, with significant cell adhesion, migration and proliferation [91]. The layered double hydroxide (LDH)/poly-dopamine (PDA) composite coating prepared on the surface of the AZ31 alloy could significantly improve the corrosion resistance of the alloy [94]. However, it was found that the LDH coating was not always superior to the single hydroxide coating. Zhang et al. [95] fabricated three kinds of hydroxide coatings with nanosheet structures, Mg(OH)_2_, Mg-Fe LDH, and FeOOH, on the magnesium alloy treated with PEO. The coatings effectively closed the micropores formed during the PEO treatment. Compared with PEO-treated magnesium alloy, the corrosion resistance and biocompatibility of the magnesium alloy with hydroxide coating was significantly enhanced, and the trend was as follows: FeOOH > Mg-Fe LDH > Mg(OH)_2_ > PEO coatings. The FeOOH coating can be used as a novel potential coating for the surface modification of magnesium alloy implants. The coatings above all can improve the corrosion resistance of magnesium alloys, but whether they can undergo the deformation of magnesium alloy stents during implantation can be suspected.

The fluoride conversion coating, which possesses a uniform and controllable thickness and relatively high density, has the potential to considerably enhance the corrosion resistance and inhibit the degradation of magnesium alloys. Mao et al. [96] prepared a uniform and compact MgF_2_ film by chemical conversion on the surface of JDBM using hydrofluoric acid (HF). The MgF_2_ film could effectively improve the corrosion resistance of JDBM, while significantly decreasing the hemolysis rate of the alloy. In order to reduce the pollution of HF to the laboratory environment, Mao et al. [97] developed an eco-friendly and simple method to prepare a nano-scale MgF_2_ film on JDBM through a chemical conversion treatment of the alloy in a 0.1 M potassium fluoride (KF) solution. The film had a uniform and dense physical structure that significantly reduced the corrosion rate. Thereafter, JDBM stents coated with MgF_2_ film were implanted into rabbit abdominal aorta, which confirmed the excellent tissue compatibility without thrombosis or restenosis. Li et al. [98] investigated the degradation and the related mechanism of the AZ31B alloy with the fluoride conversion coating. After the alloy with the fluoride conversion coating was immersed in Hank’s solution, MgF_2_ in the coating dissolved into F ions and Mg ions. Due to the low solubility of MgF_2_, Mg(OH)_2_ formed at a sluggish rate, resulting in an even coating that is resistant to corrosion. As H_2_O and Cl^−^ enter the alloy substrate, the alloy starts to degrade, leading to the formation of Mg(OH)_2_ and H_2_. The degradation of the magnesium alloy with the fluoride conversion coating proceeded gradually, migrating inward layer by layer. The HF-treated magnesium alloy stents exhibited excellent corrosion resistance without expansion compared to the bare stents. However, after stent expansion, small fragments and cracks appeared on the surface of HF-treated magnesium alloy stents, leading to an accelerated corrosion rate [56]. Cardiovascular stents are constantly subjected to cyclic loading due to heartbeats, and microcracks on the surface of the stent severely affect implantation stability. Thus, the fluoride conversion coating still could not satisfy the high requirement for the corrosion resistance of magnesium alloy stents, and further treatment is still needed to improve the corrosion resistance and biosafety of the magnesium alloy stents. The inorganic base layer has been utilized as a pretreatment coating to form a composite coating with a polymer, which can improve the corrosion resistance and biocompatibility of magnesium alloy stents.

#### 4.3.2. Outer Polymer Coating

Compared with the inner inorganic coating, polymer coatings could endow magnesium alloy stents with superior corrosion resistance and biocompatibility. At the same time, polymer coatings can serve as a drug delivery platform, fulfilling various medical functional requirements. However, when a polymer coating is prepared on the surface of the magnesium alloy directly, the corrosion medium that permeates through the polymer coating would lead to rapid corrosion of magnesium matrix and hydrogen release. It could lead to gas accumulation underneath the coating, causing the coating to crack and fail [57,99]. Therefore, magnesium alloys often need to be pretreated before the polymer coating preparation, which provides a physical barrier to the substrate and at the same time improves the bonding between the substrate and the outer polymer coating. There are a large number of polymer coatings for magnesium alloy stents, owning excellent deformability.

- Polylactic acid (PLA) coating

Polylactic acid is a hydrophobic aliphatic polyester, which is a thermoplastic, biodegradable, and biocompatible synthetic polymer with an exceptional strength and modulus. It is classified as generally recognized as safe (GRAS) by the Food and Drug Administration (FDA) of the United States and is already used in industrial packaging and many medical devices [100,101].

The PLA coating significantly reduces the degradation rate of magnesium alloy stent, thus providing radial support to the vascular wall over a 6-month period [102]. However, the bonding between PLA and the magnesium alloy is weak, and the surface of the alloy is usually treated with fluorination to improve adhesion [56]. The MgF_2_ layer on the surface of magnesium alloys is smooth and compact, but with some micropores. The preparation of PLLA coatings using ultrasonic atomization spraying could well cover these pores and provide a good physical barrier [103]. The fluoride-treated magnesium alloy stents could remain unchanged in the neutral axis direction after crimping and dilating with a balloon catheter, while the coating appeared brittle and flaky at the deformed radius. In contrast, the PLLA coating prepared outside the fluoride-treated magnesium alloy stents had a homogeneous and pinhole-free appearance on the surface and did not show cracks, even after curling and dilation. Animal experiments also showed that fluoride-treated magnesium alloy stents with the PLLA coating exhibited better corrosion resistance and longer support compared to the fluoride-treated magnesium alloy stents, while also exhibiting excellent biocompatibility [104]. The composite coating prevented the penetration of erosion ions into the magnesium matrix to improve the corrosion resistance and reduce the corrosion rate. The PLA coating could eliminate the prior porous defects through a critical remelting treatment, which significantly improved the corrosion resistance of the magnesium alloy stents [105].

**Figure 7:**
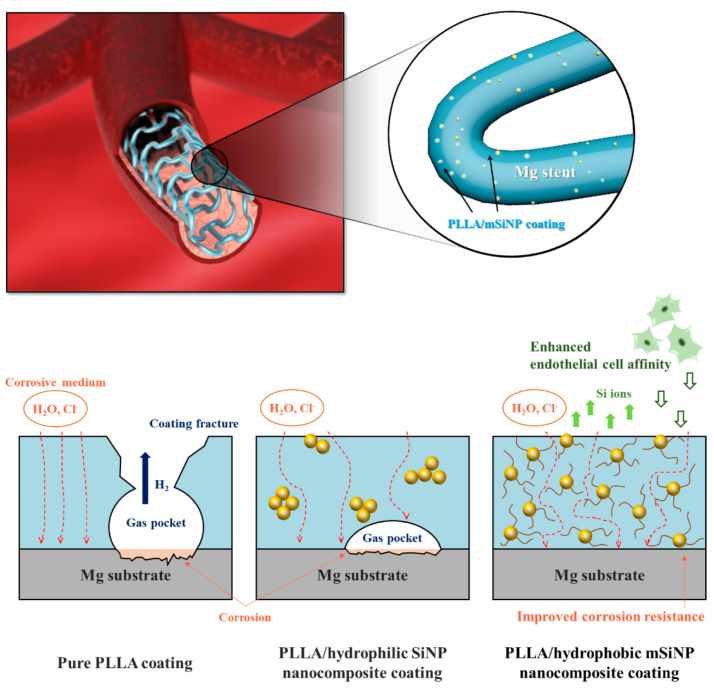
Schematic illustration of the biofunctional PLLA/mSiNP nanocomposite coating on a magnesium alloy stent [107].

It has been found that the addition of certain specific nanoparticles to PLA coatings could improve the corrosion resistance and show better biocompatibility. Shi et al. [106] added 2% Mg(OH)_2_ particles to PLLA coatings to improve protective ability of the coating. The incorporation of Mg(OH)_2_ particles decreased the hydrophobicity and enhanced the water absorption of the PLLA. As a result of polymer swelling induced by water ingress, numerous defects/channels were created in the polymer coating due to the expansion of the coating volume during the swelling process. This allowed H_2_ to diffuse more easily through the composite coating compared to the dense PLLA coating. As a result, gas pockets did not form beneath the coating, preventing it from peeling off the substrate. Taking a similar strategy, Park et al. [107] integrated silica nanoparticles that were surface-functionalized with hexadecyltrimethoxysilane (mSiNPs) into a PLLA coating (Figure 7). The mSiNPs that were exposed contributed to the hydrophobicity of the coating, which could interfere with initial water penetration; in addition, the embedded particles could extend the water transport path to increase the time delay to contact with the magnesium alloy substrate. The delay impeded the degradation of the magnesium alloy, resulting in minimal generation of H_2_ and a low level of magnesium ions released. Meanwhile, the released silicon ions are considered a driving factor in angiogenesis as they activate the synthesis of vascular endothelial growth factor (VEGF) and its receptor (VEGF receptor 2). This leads to the heightened proliferation, migration, motility and differentiation of endothelial cells.

However, the PLA coating is relatively hard and brittle, and the coating could peel and crack on the surface after the stent expands, causing serious localized corrosion and deeper pits [108].

- Poly (lactic-co-glycolic acid) (PLGA) coating

PLGA is another coating for magnesium alloys to reduce the degradation rate and enhance cell adhesion [109], which is approved by the FDA and European Medicine Agency (EMA) in various drug delivery systems for humans [110].

As a single coating, PLGA may not effectively improve the corrosion resistance of the magnesium alloy as expected, which is related to the bulk erosion of PLGA [111]. As corrosion medium easily diffuses into and through the PLGA coating, both PLGA and the magnesium alloy begin to degrade simultaneously. Once bulk erosion has started, by-products of polymer degradation can react with the corroded magnesium ions or magnesium hydroxide, forming soluble magnesium lactates or magnesium glycolates. These can prevent the formation and growth of a dense and thick layer of magnesium hydroxide, and the polymer may provide little or no protection for the following time [99]. When the PLGA coating was applied to a magnesium alloy stent alone, there were several wrinkles, creases and partial detachment that appeared after expansion process, which could not provide good protection for the stent [112].

- Polycaprolactone (PCL) coating

Polycaprolactone is an aliphatic polyester consisting of hexanoic repeat units. Its broad applications and intriguing attributes, including controlled degradability, miscibility with other polymers, biocompatibility, and potential use of monomers derived from renewable sources, render it a highly valuable polymer [113]. PCL inhibits gas evolution on the base metal and is a promising candidate as a coating material for controlling the degradation rate and mechanical strength of magnesium alloys [100]. PCL has been approved by FDA for use in a wide range of biomedical products, such as drug delivery, bone graft substitution, and tissue engineering applications [114,115,116].

The glass transition temperature (T_g_) of PCL was measured using differential scanning calorimetry (DSC) to be (−64.5 ± 3.9 °C) [108], which implies that the PCL coating is in a rubbery and flexible state in the biological environment, and the coating maintains its macroscopic integrity after deformation of the stent and does not undergo localized corrosion. The dense coating exerts a long-lasting decelerating impact on corrosion by establishing diffusion barriers and autoinhibition of the corrosion process [117]. Compared to the PLA-coated high purity magnesium (HPM), the PCL-coated HPM showed a higher *E*_corr_ and lower *I*_corr_ [57]. The PCL coating could improve the cell adhesion and tissue growth around the magnesium alloy implant by decreasing its corrosion rate [116,118].

- Poly (trimethylene carbonate) (PTMC) coating

PTMC is a popular soft material used in scaffold applications for the regeneration of soft tissue, as well as a hydrophobic segment in amphiphilic block copolymers for drug delivery [119]. The degradation process of PTMC is slower than that of PLLA and other aliphatic polyesters. A study showed that enzymatic degradation played a crucial role in the surface erosion [120]. Meanwhile, PTMC possesses elasticity and softness, and so it can be used as a surface coating material for magnesium alloy stents.

**Figure 8:**
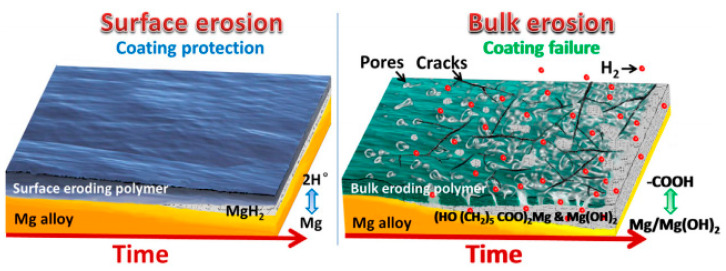
Schematic diagram of surface and bulk erosions [121].

It has been found that a uniform thin PTMC coating on magnesium alloys eroded from the surface to the interior when exposed to the biological environments [121]. As a result, it created a protective pathway that impeded electrolyte diffusion from the blood to the magnesium alloy, thus minimizing substrate corrosion. PTMC hydrolysis is a nearly neutral ionic process and maintains a physiological pH during degradation. PTMC allows a minimal amount of electrolyte penetration through the coating to interact with the magnesium alloy substrate beneath. The remaining PTMC preserves the stability of this thin corrosion layer, regardless of whether the product layer is Mg(OH)_2_ or MgH_2_, to prevent further corrosion and dissolution (Figure 8). The addition of graphene oxide (GO) into PTMC coatings has the potential to enhance the water barrier efficacy of composite coatings. This improvement is due to the two-dimensional GO structure, which elongates the solution’s penetration path through the labyrinth effect, and subsequently restrains hydrogen accumulation beneath the polymer layer, leading to better corrosion resistance [122,123].

PTMC coated samples showed good cytocompatibility and hemocompatibility, with very few platelets adhering on the surface. Compared with PLGA, PTMC coatings have a more stable and sustained drug release capacity and can inhibit the proliferative of HUVSMCs for a long period of time due to the much slower surface erosion behavior and degradation rate [124]. Atorvastatin calcium (ATVC) was loaded into the PTMC delivery coating on the magnesium alloy surface, which was able to promote the rapid endothelialization of HUVECs and regulate growth of HUVSMCs, further preventing endothelial hyperplasia and inflammatory responses [125]. However, PTMC lacks functional groups, which limits further functional modifications, for example, the conjunction of bioactive components and immobilization on metal surfaces. Chen et al. [126] developed amino-grafted PTMC polymers that were immobilized on the surface of magnesium alloys through the reaction of amino and carboxyl groups, and the immobilized polymeric coatings could potentially offer improved resistance to detachment during clinical delivery processes, including stent dilatation.

- Polyurethane (PU) coating

Polyurethane is an elastic polymer. The polar urea groups in polyurethane urea enhance hydrogen bonding in the hard segments, which act as strong physical crosslinkers. Solid polyurethane coatings possess good elastomeric mechanical properties due to their high molecular weight, low crystallinity and low glass transition temperature (Tg < −46 °C) [127], making them a potentially biodegradable polymer coating for magnesium alloy stents. Gu et al. [128] investigated hemocompatibility, the dynamic degradation behavior and drug release of poly(carbonate urethane) urea (PCUU) and poly(ester urethane) urea (PEUU) coatings on magnesium alloy stents. Compared with PEUU-coated, PLGA-coated and bare magnesium alloy stents, the PCUU-coated stents showed better corrosion resistance and reduced thrombotic deposition. Compared to the PLGA coating, Arg-PEUU and Arg-Leu-PEUU have better bonding to magnesium alloys while exhibiting better corrosion resistance and biocompatibility [129,130]. The advantage of corrosion resistance could be attributed to the surface degradation nature of the amino acid based polyester urea urethane family [131], which resulted in a slow degradation rate in simulated body fluid. Arg-PEUU and Arg-Leu-PEUU coatings reduced both platelet adhesion and the hemolysis rate, had better cell adhesion to HUVECs, stimulated NO release from HUVECs, and had the ability to delay thrombosis and restenosis.

- Silane coating

**Figure 9:**
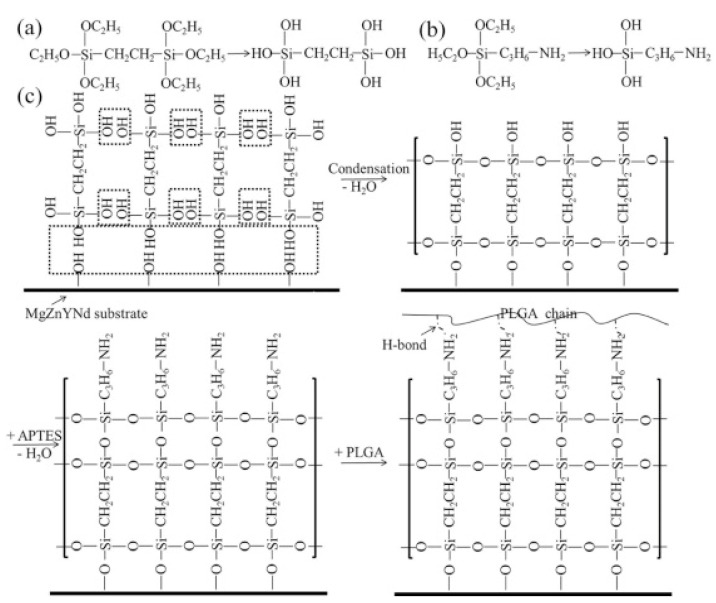
Chemical structure of BTSE (**a**) and APTES (**b**); (**c**) surface modification procedure on the ZE21B alloy [133].

Silane coatings for magnesium alloys have been found to be valid, ecofriendly, and economical. Liu et al. [132] investigated a one-step reaction in which a cross-linked 3-amino-propyltrimethoxysilane (APTES) silane physical barrier layer was introduced onto the surface of a ZE21B alloy prior to electrostatic spraying of the rapamycin-eluting PLGA coating. Solid polysiloxane networks with exposed amine functional groups were formed by in situ APTES polycondensation, providing an effective physical barrier and strong bonding function. The APTES-treated magnesium alloy showed very favorable compatibility with HUVECs and HUVSMCs. Animal experiments confirmed that APTES-treated Mg-Zn-Y-Nd stents implanted into porcine coronary arteries for 6 months showed excellent tissue compatibility and re-endothelialization capacity without severe signs of injury, thrombosis, or restenosis of the vascular wall. After that, a simple two-step reaction was used to introduce anticorrosive silane pre-treatment on the ZE21B alloy before coating with PLGA [133] (Figure 9). The first step was to immerse the NaOH-activated ZE21B alloy in bistriethoxysilylethane (BTSE) to form a cross-linked silane coating layer with enhanced corrosion resistance, and the second step was to treat the BTSE-modified ZE21B alloy with APTES to immobilize the amino functional groups to form hydrogen bonds with the outer PLGA coating. Compared to the APTES pretreatment, the cross-linked bilayer BTSE-APTES pretreatment showed better corrosion protection and biocompatibility.

There have been a large number of research studies on polymer coatings prepared on magnesium alloy stents that indicated that each polymer with its own characteristics was hard to meet the clinical requirements of magnesium alloy stents with surface protection separately. We should make full use of the performance advantages of various coatings to integrate magnesium alloy stents with excellent corrosion resistance. On this basis, new protective strategies should be sought to further improve the clinical safety and effectiveness of magnesium alloy stents.

## 5. Research Trends and Outlook of Magnesium Alloy Stents

### 5.1. Research Trends of Magnesium Alloy Stents

**Figure 10:**
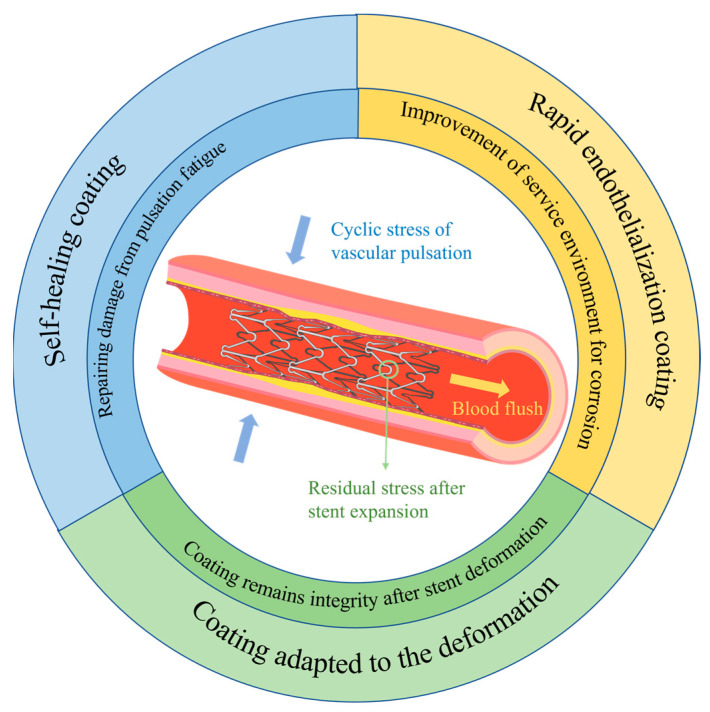
Further exploration and study of magnesium alloy stents.

Based on the corrosion mechanism of magnesium alloy stents, together with their deformation during usage and features of the service environment, further comprehensive exploration and studies are necessary on magnesium alloy stents. This calls for adapting coatings to the deformation of the stents, preparing rapid endothelialization coatings to enhance the service environment of the stent, and constructing coatings with self-healing functions, with the aim of developing bioabsorbable magnesium alloy stents with controlled degradation (Figure 10).

#### 5.1.1. Study of Coatings Adapted to the Deformation Mechanics of Stents

Biodegradable magnesium alloy stents are currently delivered mainly through balloon-expandable catheter systems during implantation. However, the surface coating of the stent may crack or even peel off from the substrate due to deformation after balloon expansion, indicating that the coating is not compatible with the deformation mechanics of the stent. The incompatibility of the surface coating with magnesium alloy stents may expedite the local corrosion of the stent platform. Consequently, the corrosion of the stent quickens, leading to the speedy loss of radial support. Thus, a coating that aligns with the deformation mechanics of the stent is crucial.

**Figure 11:**
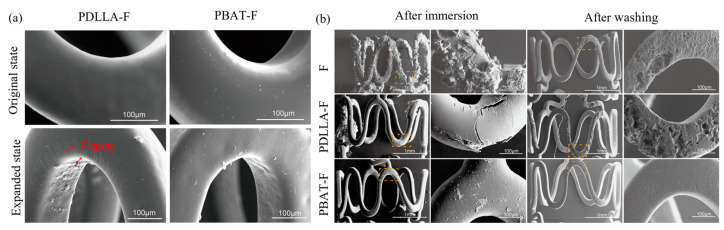
Surface morphologies of stents with PDLLA-F and PBAT-F coatings after deformation (**a**) and immersion in PBS (**b**) [135].

In order to reduce the stress concentration distribution of the stent after deformation, the design of the magnesium alloy stent should be optimized using the FEA method. Furthermore, it is essential to select a polymer with higher elasticity that is specifically tailored to the deformation of magnesium alloy stent when creating the coating. This can prevent damage to the coating caused by the deformation of the stent, which will reduce the corrosion rate of magnesium alloy stent. In addition, it is crucial to pay a close attention to the corrosion pattern of the polymer coating and the bonding strength between the substrate and the polymer coating. Shi et al. [134] coated magnesium alloy discs and stents with a relatively high plasticity polymer, poly(butyl acrylate) (PBA), through a dip-coating method and ultrasonic spraying system, respectively. Compared with poly-D,L-lactic acid(PDLLA), the PBA polymer coating exhibited excellent mechanical properties without any noticeable damage during the expansion of the stent, indicating that this coating has the ability to withstand deformation. The PBA coating maintained the structural integrity of the magnesium alloy stent with slight corrosion after 21 days of service. Thereafter, poly(butylene-co-adipate-terephthalate) (PBAT) was selected for the reliable protection of HF-treated magnesium alloys, due to its high elongation at break [135]. The PBAT polymer coating is more suitable for the surface protection of magnesium alloy stents due to its excellent ability to hinder electrolyte permeation and withstand complex deformation. In the long term, the PBAT-F coating showed significantly better properties than the PDLLA coating, and the magnesium alloy stent with the PBAT-F coating showed homogeneous corrosion, as shown in Figure 11.

A quantitative study of the compatibility of coatings on the stents with deformation should be further carried out. The finite element simulation method has been used to optimize the design of stents, which should be expansively used to establish a model of magnesium alloy stents with coatings. Based on it, the effects of compression and expansion on the coating integrity and binding performance of the coating on the surface of the stent need to be studied. These can provide the theoretical basis and data support for screening coatings for magnesium alloy stents, which is an important research direction in the future.

#### 5.1.2. Rapid Endothelialization Coating on Magnesium Alloy Stents

Most traditional methods to decrease the corrosion rate of magnesium alloy stents have been concentrated on enhancing the corrosion resistance of the stent material or the property of the coating. However, less attention has been paid to the influence of the implantation surroundings on the degradation rate of magnesium alloy stents. A strategy is proposed like this: the more rapidly the stent is covered by the endothelium post-implantation, the lower the incidence of restenosis and thrombosis that occur on the stent. Furthermore, the nascent endothelium quickly covers the magnesium alloy stents after implantation, shortening the cycles of flushing by blood. This also results in a change from the initial-stage blood corrosion to the tissue–fluid exchange corrosion, further reducing the corrosion rate of the magnesium alloy stents from the perspective of improving the in vivo service environment.

Conventional medications carried on the surface of stent, such as rapamycin, paclitaxel and sirolimus, are utilized to inhibit in-stent restenosis. However, they have inhibitory effects on both endothelial cells and smooth muscle cells, which can lead to delayed endothelialization and a prolonged exposure time of the stent in the blood. Thus, the development of a functional coating with the properties of enhancing endothelial cell proliferation and suppressing smooth muscle cell proliferation is a crucial area for the future research of magnesium alloy stents.

PDA coatings can be used directly to control the behavior of cells on the stent surface and are able to solve the problems associated with re-endothelialization and restenosis of the stents. It can enhance the attachment and proliferation of human umbilical vein endothelial cells (HUVECs) [136,137]. In addition, the PDA coating has the ability to effectively inhibit the adhesion and proliferation of human umbilical vascular smooth muscle cells (HUVSMCs) [138]. Arg-Glu-Asp-Val (REDV) is a fibronectin-derived peptide, and when synthetic peptides containing this sequence are immobilized on otherwise cell non-adhesive substrates, endothelial cells adhere and spread, but fibroblasts, vascular smooth muscle cells and platelets do not [139]. Currently, studies related to the preparation of functionalized coatings on magnesium alloys containing REDV have been carried out [140,141,142]. However, the current studies are mainly focused on in vitro studies, and animal experiments of magnesium alloy stents with functionalized REDV coatings should be the next research focus.

**Figure 12:**
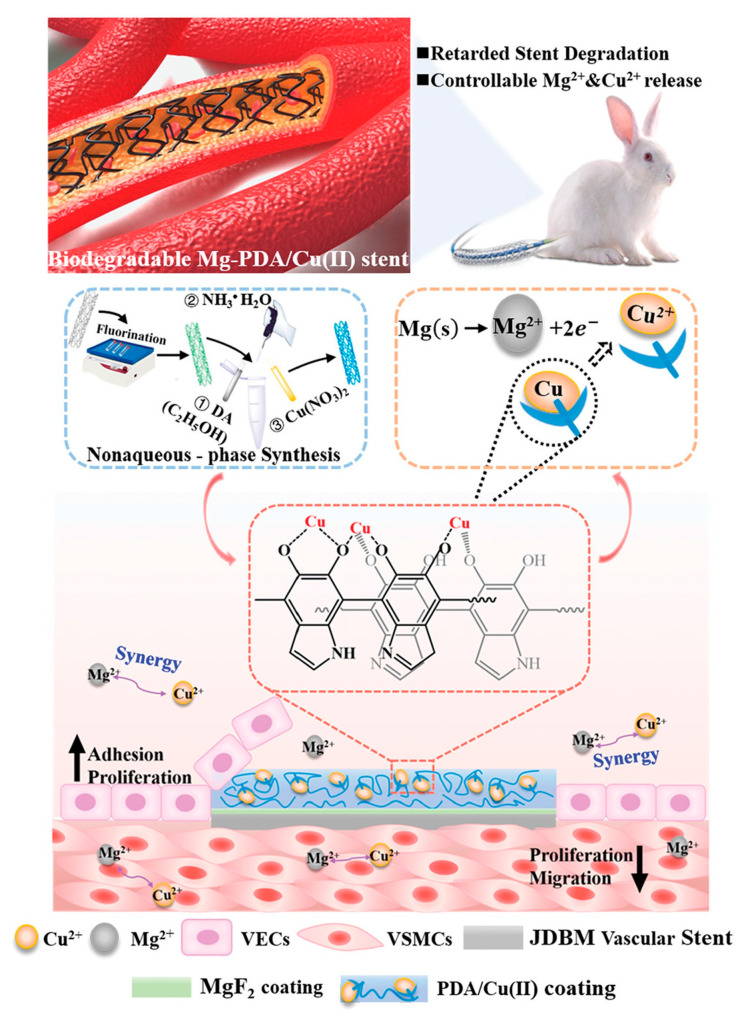
Schematic illustration of the PDA/Cu^2+^-eluting coating on magnesium alloy stents for controlling the degradation of the Mg matrix and modulating the local release of Mg^2+^ and Cu^2+^, which contributed to the superior bio-efficacy with a prolonged stent durability, and enhanced anti-inflammatory and anti-restenosis functions through synergistic effects on fostering HUVECs while inhibiting HUVSMCs [145].

The copper (Cu)-bearing metals (including 316 L stainless steel and cobalt-based alloys) developed by Ke Yang’s team at the Institute of Metal Research, Chinese Academy of Sciences, exhibited excellent properties of promoting endothelialization and inhibiting smooth muscle cell proliferation and thrombosis as a novel platform material for future vascular stents [143]. Inspired by this, the copper-containing coating was further investigated [144], and the preliminary results showed excellent biofunctional properties, as the implanted magnesium alloy stent with the copper-loaded coating was covered by endothelium within 7 days, indicating that the copper-loaded coated magnesium alloy has an application prospect. Based on the same strategy, Li et al. [145] developed a facile copper-incorporated coating system through the nonaqueous phase synthesis of PDA to facilitate Cu^2+^ capture along with a robust film deposited on easily corrodible magnesium, which subsequently enabled sustained Cu^2+^ elution (Figure 12). The generated Cu^2+^-integrated Mg remarkably promoted HUVEC growth and endogenetic NO release, while simultaneously inhibited HUVSMCs. In vivo implantation studies further confirmed that the Cu^2+^ released from the magnesium alloy stent could improve stenting durability, promote rapid re-endothelialization, and alleviate neointimal hyperplasia, without obvious injury, inflammation, or thrombosis.

The potential biofunctional coatings with superb pro-endothelial effects should be further explored, which should be suitable for magnesium alloy stents to promote biocompatibility and biosafety.

#### 5.1.3. Introduction of Self-Healing Mechanism into Coatings on Magnesium Alloy Stents

The corrosion fracture of magnesium alloy stents is essentially attributed to the stress corrosion and fatigue corrosion of the magnesium alloy. According to the characteristics of the use and service process of magnesium alloy stents, stress, deformation and damage of the stents are inevitable. If a material with a self-healing property is introduced into the magnesium alloy or the coating, the damage formed during the service process of the stent can be repaired in real time, thus reducing the degradation rate of the magnesium alloy stent and prolongating the fracture cycle of the stent. It is a promising surface protection strategy for magnesium alloy stents. How to construct a self-healing coating on surface of magnesium alloy stents is another research direction in the future.

### 5.2. Outlook

The magnesium alloy stent is one of the three types of biodegradable metal stents developed at present, and it is the earliest biodegradable metal stent applied in the clinic. Clinical results have shown long-term biosafety, but the physical support performance of the stent in the acute stage after surgery is not stable enough. It is necessary to further optimize the mechanical properties of the stent platform, the short-term protective effect of the coating, the rapid endothelialization function of the endothelialization-promoting coating, and the self-healing function in the stent so as to realize the effective support of magnesium alloy stents at the initial stage of implantation and the gradual loss of support after 3–6 months of implantation, that is, controllable degradation.

## Data Availability

Not applicable.
